# A Mechanical Structure Design and Simulation-Based Validation of a Novel Compact and Low-Cost 3-DOF Robotic Arm

**DOI:** 10.3390/s25237356

**Published:** 2025-12-03

**Authors:** Jiahe Chen, Bojun Jiang, Shu Zhu, Jun Wang

**Affiliations:** 1 Wenzhou Key Laboratory of Al Agents for Agriculture, Wenzhou 325006, China; 2 Wenzhou Vocational College of Science and Technology, Wenzhou 325006, China; fut.streich@outlook.com; 3 Jiangsu Key Laboratory of 3D Printing Equipment and Manufacturing, School of Electrical and Automation Engineering, Nanjing Normal University, Nanjing 210023, China; 13013912211@163.com

**Keywords:** robotic arm, 3-DOF, skeleton modeling, compact structure

## Abstract

This paper presents the mechanical design and simulation-based validation of a novel compact and low-cost 3-DOF dual-arm robotic system tailored for space-constrained applications such as rescue robotics. The proposed design achieves a fully folded footprint of 366 × 226.3 × 100 mm through an orthogonal joint configuration and modular structure, while maintaining a hemispherical workspace for each arm. Key innovations include the following: (1) A cost-optimized architecture with only 3 motors per arm (total system cost ~£2000), enabled by hybrid manufacturing (laser-cut acrylic hull and 3D-printed ASA-CF reinforced links with 3740 MPa flexural modulus); (2) a custom Python-based skeleton modeling tool that automates D-H parameter generation and kinematic analysis, supporting rapid design iteration; (3) verified collision-free operation via point cloud analysis, demonstrating successful target grasping (50 mm objects) and dual-arm coordination despite a 5–20 mm deflection tolerance. The system addresses critical limitations in affordable, deployable manipulators, with future work focusing on 3D printing and part manufacturing in industry applications.

## 1. Introduction

The robotic arm is a class of manipulators that is commonly employed in diverse industrial scenarios. The operation of the system is based on the change in position of objects between specified spatial coordinates, a process informed by predefined positional data. The configuration of such systems can be set up in one of two ways: either manually or autonomously. Furthermore, the systems are adaptable to both stationary and mobile platforms [1]. Robotic manipulation has been explored since the start of robotics research [2]. Despite significant advancements in robotic manipulation, current robotic arms remain unsuitable for widespread domestic deployment [3]. The majority of commercially available systems are prohibitively expensive, often exceeding 20,000 USD [4], and are typically heavy, with weights surpassing 10 kg. Furthermore, these manipulators require permanent mounting to fixed structures or the use of bulky mechanical clamps for base stabilization, thereby limiting their mobility and making relocation cumbersome once installed [5].

Numerous studies have been undertaken to mitigate the aforementioned limitations, with a significant body of research focusing on the development of mobile manipulators [6,7], a robotic system that integrate mobility and manipulation capabilities to enhance versatility and adaptability across diverse environments. In the field of lightweight robotic arm design, Gutiérrez et al. investigates the manufacturing process of lightweight robotic arm components [8], with each individual part requiring 6~10 h of fabrication. All components were first designed through three-dimensional simulation and subsequently manufactured using 3D printing technology. The resulting robotic arm meets the specified performance requirements in terms of raw material selection, metallic reinforcement structures, maintainability, and flexible coupling design. In addition, a low-cost robotic arm incorporating a counterbalance mechanism was developed to maintain stability during operation [9]. The proposed mechanism effectively reduces the robot’s movement torque, thereby enabling the use of relatively low-cost motor components [10].

The above designs are all centered around a single goal: increasing the mobility of robotic arm installations, reducing the cost, and reducing the size of the robotic arm to suit non-industrial applications. In this work, a lightweight and low-cost 3-DOF robotic arm was developed for meeting the above needs. This paper will explore and study the design of a lightweight robotic arm that utilizes a modular installation concept, enabling the robotic arm to be stored in a container of a specific size and shape when retracted, thereby reducing the dependence of specific installation environments. Additionally, this paper will also investigate issues related to the working space, control, and trajectory planning of this robotic arm design.

Specifically, this paper firstly presents the implementation background of this work. The author examines the applications of robotic arms in industrial production, personal use, and research-related scenarios, and reviews various robotic arm designs currently available in the market as well as those reported in the research domain. Based on a comprehensive analysis and comparison of different designs, the author identifies limitations in existing robotic arm configurations, offering valuable guidance for defining the design objectives of this type of robotic arm. Subsequently, this work conducts an in-depth study on the design of the robotic arm from the ground up. Starting with the defined task objectives, the author first examines the overall structural design of the dual-arm configuration, discussing how the structural layout can meet all task requirements. Then, we investigate commonly used manufacturing approaches in related fields that are suitable for this project and finalizes both the fabrication process and the materials to be used for producing physical robotic arms. Thirdly, a simple simulation tool was developed for assessing the applicability of the design of our robotic arm. Then, a thorough mathematical study and discussion of the underlying principles of the simulation tool was conducted, accompanied by an explanation of its implementation at the code level.

## 2. Mechanical Structure Design of the 3-DOF Robotic Arm

This work aims to develop a compact robotic arm, which necessitates constraints on its minimum dimensions in the fully folded state. The target outcome of this work is a dual-arm robotic system capable of performing the following functions: automatically detecting targets with specified features using a camera; grasping the target with a single arm; coordinating both arms to transfer the target from one arm to the other. In order to meet the requirements of high flexibility, high compactness, and low cost of this robotic arm, our robotic arm can be flexibly folded in a prismatic volume, and its minimum dimensions of in the fully folded state was 366 × 226.3 × 100 mm. In addition, the cost for our robotic arm is approximately £2000.

### 2.1. Overall Structure Design

**Figure 1:**
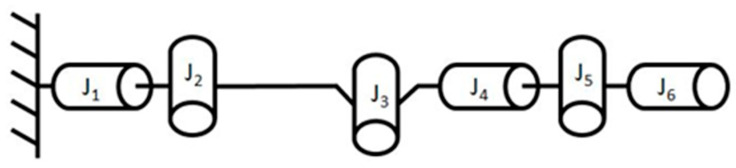
Joints configuration proposed in the related paper [12].

The first challenge encountered in achieving the current task objectives was how to design a solution that would allow the entire robotic arm, which meets the requirements, to be stored in a container of the target size when retracted and to be extended for operation. In this work, we found inspiration from another paper in a related field [11]. The study proposed a compact and cost-effective small-scale robotic arm design that closely aligns with the requirements of this project. In the proposed approach, the rotation axes of adjacent joints are arranged orthogonally, a configuration that effectively addresses the objective of sample grasping within a three-dimensional workspace [12], as illustrated in Figure 1.

**Figure 2:**
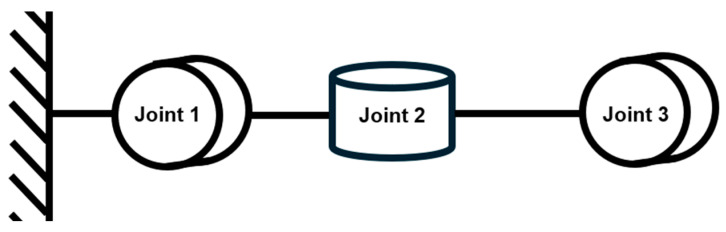
Joints configuration applied in our project.

After confirming the overall joint configuration design, additional project-specific constraints, such as budget and manufacturing conditions, must be taken into account. A survey of mainstream suppliers of motors for robotic arms revealed that the price of a brushless DC motor together with its motor control board is typically around £200. As this project aims to enable two robotic arms to perform a handshake and sample exchange, a symmetrical pair of arms is required. Following the design presented in the aforementioned paper, the number of motors per arm would reach six, resulting in a total of twelve motors significantly exceeding the project budget and contradicting the original objective of developing an “economical” robotic arm. Consequently, after careful consideration, the project team proposed an improved design in which the number of motors, and thus joints, in each arm is reduced to three (Figure 2). It must be acknowledged that, constrained by conditions mentioned earlier, this design is essentially a degenerated version of the six-axis single-arm robotic manipulator. With the reduction in the number of motors per arm, the degrees of freedom of each arm are limited to three. In other words, this configuration can only control the spatial position of the end-effector attached to the link following Joint 3, without determining its orientation.

### 2.2. Manufacturing of the Robotic Arm

Given the total project budget of £2000, the selection of manufacturing methods must balance cost-effectiveness, achievable precision, available materials, and suitability for small-scale project implementation. After reviewing the relevant materials, we are acknowledged that common fabrication approaches for small-scale engineering projects include CNC machining, laser cutting, and additive manufacturing (3D printing).

**Figure 3:**
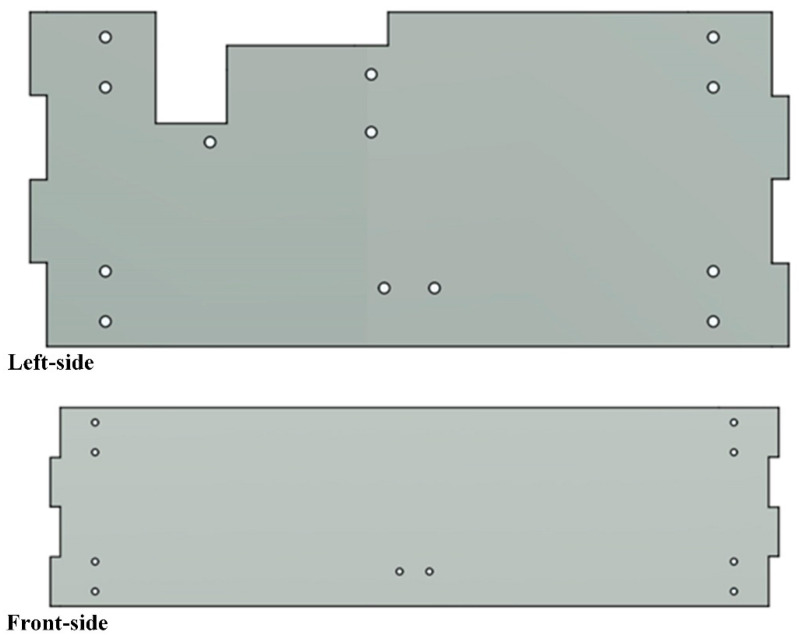
Left-side and front-side of the hull.

According to the comparison of various manufacturing methods (Table 1), for the convenience of manufacturing and cost control, different manufacturing methods are used for different components. The hull design comprises multiple interlocking panels with minimal protrusions (Figure 3), making it ideally suited for 2D planar cutting and subsequent assembly. For the robotic arm body, FDM 3D printing will be employed, allowing for the fabrication of complex joint housings and link geometries without the need for expensive tooling. These methods also facilitate rapid prototyping and iterative refinement, which is advantageous for meeting the mechanical and functional requirements of the project while maintaining budgetary limits. This hybrid manufacturing approach maximizes cost-effectiveness and functional performance while remaining within the £2000 project budget.

### 2.3. Material Selection

In additive manufacturing (3D printing) for the robotic arm body, material selection plays a critical role owing to requirements for rigidity, dimensional stability, and resistance to bending. Commonly used 3D printing materials include PLA, ABS, PETG, ASA, and their reinforced variants. The table below (Table 2) provides the basic printing properties of common 3D printing materials [13,14,15].

In addition to the base materials, carbon-fiber-reinforced variants of these materials are also available. The -CF (carbon-fiber-reinforced) versions incorporate short carbon fibers into the polymer matrix, significantly enhancing stiffness, dimensional stability, and strength-to-weight ratio compared to their unreinforced counterparts [16,17,18]. These properties make -CF variants particularly suitable for applications requiring high rigidity, lightweight structures, and stability under mechanical load. Given the project’s requirement for adequate resistance to bending and deformation, ASA-CF stands out due to its superior stiffness, UV/weather resistance, and minimal warping. These properties make it particularly suitable for load-bearing, compact robotic arm structures. The mechanical properties of ASA-CF materials are shown in Table 3 [19,20].

### 2.4. Motor Selection of the Robotic Arm

In this subsection, we will examine the motor selection process for each joint. Determining the appropriate motors prior to the overall 3D modeling of the robotic arm is crucial, as it directly influences the design of the connection structures at the joints. Moreover, the overall structural configuration of the robotic arm, in turn, affects the required specifications of each motor, such as output torque and other performance parameters.

**Figure 4:**
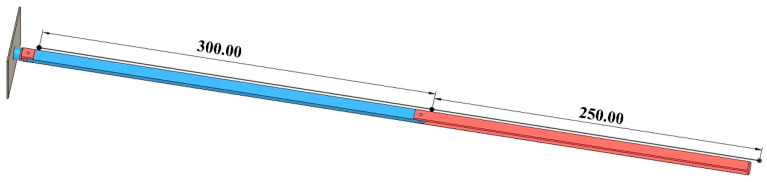
Schematic diagram of robotic arms.

Based on the joint configuration design, a simplified three-dimensional schematic model was constructed in Fusion, as illustrated in Figure 4.

Each robotic arm has three degrees of freedom, and each joint is a rotary joint. Starting from the direction of connection to the satellite body, the components are named Joint 1, Link 1, Joint 2, Link 2, Joint 3, and Link 3 in sequence. The end-effector will be mounted at the distal end of Link 3 in future implementation. Considering the joint configuration design mentioned earlier and the project requirements, namely that the entire robotic arm must be folded within a space of 366 × 226.3 × 100 mm, Link 2 is approximately 300 mm in length, and Link 3 is approximately 250 mm. Under ideal conditions, the length of Link 1 should be minimized and is therefore neglected in the theoretical calculations in this section; however, it will be taken into account in the subsequent practical design.

The initial goal of the robotic arm’s gripping function is to grasp a 0.1 kg object moving at an acceleration of 0.05 m/s^2^ in a gravity environment on the earth. To simplify the computational process, the authors will first calculate the required torque for each joint motor of the equivalent target in a zero-gravity environment, and subsequently account for the effects of gravity. According to some related research, the general order of magnitude is that the capability to lift 2 kg on Earth corresponds to moving approximately 1000 kg in space [21,22,23]. Therefore, to meet the design dimensions for this project in a space environment, and taking into account a safety margin, the design specifications in an ideal zero-gravity environment were adjusted to a payload capacity of 20 kg and an acceleration of 0.05 m/s^2^.

As is known from basic physics, when the distance between the center of mass and the axis of rotation of a system is greater, and the angular acceleration (linear acceleration) of the centre of mass is faster, the rotational inertia of the system is greater. Therefore, when the robotic arm model is fully extended, the torque output requirements for each joint are at their maximum. The following analysis examines the torque output requirements for each joint under these conditions. Moreover, as the left and right robotic arms are structurally identical, the following computational analysis is presented using the left arm as a representative example.

Assume the following:

$m_{i}$—mass of the motor at joint i, $i\;\in \;\left[1,\;3\right]$;
$m_{o}$—mass of the integrity of end-effector and target object in space environment;
$l_{i}$—length of link i, $i\;\in \;\left[1,\;3\right]$;
S—cross-sectional area of the link;
ρ—density of the link material;
a—target linear acceleration;
α—target angular acceleration.

For joint 1:

As is mentioned earlier, since the distance between joint 1 and joint 2 is very small relative to the size of the robotic arm, the length of the link 1 can be neglected. Additionally, since the motor on joint 1 is rigidly mounted to the hull framework, its mass can therefore be neglected when calculating the maximum required output torque of the motor. Since the distance between joint 1 and joint 2 is considered negligible in the theoretical calculation, the maximum theoretical output torque required for the motors on these two joints is therefore identical, which occurs when link 1, link 2, and link 3 are fully extended. Assume $\tau_{1}$ as the maximum theoretical output torque on joint 1, we have

$$\tau_{1}=I_{1}\alpha_{1}$$

$I_{1}$ represents the total moment of inertia of all components rotating about joint 1, including link 1 (neglected), link 2, link 3, joint 2 (neglected), joint 3, and the end-effector–target assembly, summed with respect to the rotation axis of joint 1:

$$I_{1}=I_{link2}+I_{link3}\;+I_{motor3}+I_{object}$$

$$I_{link2}+I_{link3}=\frac{1}{3}ML^{2}=\frac{1}{3}S(l_{2}+l_{3})\rho {\left(l_{2}+l_{3}\right)}^{2}$$

$$I_{motor3}=m_{3}{l_{2}}^{2}$$

$$I_{object}=m_{o}{{(l}_{2}+l_{3})}^{2}$$

$$\alpha_{1}=\frac{a}{l_{2}+l_{3}}$$

Therefore,

$$\tau_{1}=\left[\frac{1}{3}S\rho {\left(l_{2}+l_{3}\right)}^{3}+m_{3}{l_{2}}^{2}+m_{o}{{(l}_{2}+l_{3})}^{2}\right]\frac{a}{l_{2}+l_{3}}\;=\frac{1}{11}\left(\frac{1331}{24000}S\rho +\frac{9}{100}m_{3}\right)+\frac{11}{20}\;\;\;\;\;\;$$

Similarly, for joint 2,

$$\tau_{2}=I_{2}\alpha_{2}$$

$$I_{2}=I_{link2}+I_{link3}+I_{motor3}+I_{object}$$

$$I_{link2}+I_{link3}=\frac{1}{3}ML^{2}=\frac{1}{3}S(l_{2}+l_{3})\rho {\left(l_{2}+l_{3}\right)}^{2}$$

$$I_{motor3}=m_{3}{l_{2}}^{2}$$

$$I_{object}=m_{o}{{(l}_{2}+l_{3})}^{2}$$

$$\alpha_{2}=\frac{a}{l_{2}+l_{3}}$$

Therefore,

$$\tau_{2}=\left[\frac{1}{3}S\rho {\left(l_{2}+l_{3}\right)}^{3}+m_{3}{l_{2}}^{2}+m_{o}{{(l}_{2}+l_{3})}^{2}\right]\frac{a}{l_{2}+l_{3}}\;=\frac{1}{11}\left(\frac{1331}{24000}S\rho +\frac{9}{100}m_{3}\right)+\frac{11}{20}=\tau_{1}\;\;\;\;$$

For joint 3, things have changed somewhat:

$$\tau_{3}=I_{3}\alpha_{3}$$

$$I_{3}=I_{link3}+I_{object}$$

$$I_{link3}=\frac{1}{3}ML^{2}=\frac{1}{3}Sl_{3}\rho {l_{3}}^{2}$$

$$I_{object}=m_{o}{l_{3}}^{2}$$

$$\alpha_{3}=\frac{a}{l_{3}}$$

Therefore,

$$\tau_{3}=(\frac{1}{3}S\rho {l_{3}}^{3}+m_{o}{l_{3}}^{2})\frac{a}{l_{3}}\;=\frac{1}{960}S\rho +\frac{1}{4}\;\;\;\;$$

where the units of $\tau_{1}$, $\tau_{2}$, and $\tau_{3}$ are N·m.

Now substitute the estimated values into the calculation. Considering that we have preliminarily decided to use ASA-CF material for 3D printing to manufacture robotic arms, ρ is taken as $1200\;kg/m^{3}$. Based on the preliminary design draft and the motor parameter documentation for similar sizes available on the market, the link is designed as a square prism with a cross-section of 9 mm per side, resulting in a cross-sectional area of $81\;{mm}^{2}$ (i.e., $8.1\times 10^{-5}\;m^{2}$). So $S=8.1\times 10^{-5}\;m^{2}\;$. The average mass of commercially available motors of this size is approximately 100 g; therefore $m_{i}=0.1\;kg$ are taken, leading to the following further conclusions:

$$\tau_{1}=\tau_{2}\approx 0.5512\;N\cdot m=5.6210\;kg\cdot cm$$

$$\tau_{3}\approx 0.2501\;N\cdot m=2.5502\;kg\cdot cm$$

**Figure 5:**
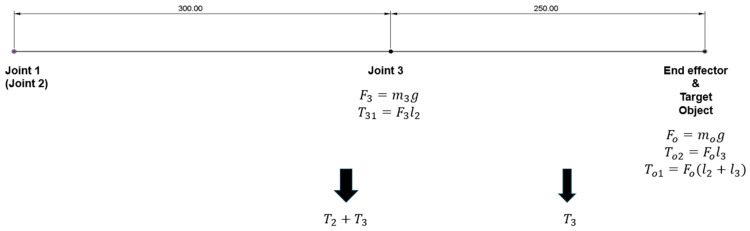
Force analysis diagram of the links in the fully extended status, taking earth gravity into account.

However, the foregoing results neglect gravity. To determine the maximum required output torque at each joint, the gravitational loading of all links and joints must also be accounted for. The calculation process is basically the same as that in a zero-gravity environment, with the difference being that the torque provided by gravity needs to be added (Figure 5).

$$\tau_{1}^{\prime }=\left[\frac{1}{3}S\rho {\left(l_{1}+l_{2}\right)}^{3}+m_{3}{l_{1}}^{2}+m_{o}^{\prime }{{(l}_{1}+l_{1})}^{2}\right]\frac{a}{l_{1}+l_{1}}+T_{31}+T_{o1}+T_{2}+T_{3}$$

$$\tau_{2}^{\prime }=\left[\frac{1}{3}S\rho {\left(l_{1}+l_{2}\right)}^{3}+m_{3}{l_{1}}^{2}+m_{o}^{\prime }{{(l}_{1}+l_{1})}^{2}\right]\frac{a}{l_{1}+l_{1}}+T_{31}+T_{o1}+T_{2}+T_{3}$$

$$\tau_{3}^{\prime }=(\frac{1}{3}S\rho {l_{3}}^{3}+m_{o}^{\prime }{l_{3}}^{2})\frac{a}{l_{3}}+T_{o2}+T_{3}$$

$$T_{31}=m_{3}gl_{2}=0.2942\;N\cdot m$$

$$T_{o1}=m_{o}^{\prime }g(l_{1}+l_{2})=0.5394\;N\cdot m$$

$$T_{o2}=m_{o}^{\prime }gl_{2}=0.2452\;N\cdot m$$

$$T_{2}+T_{3}=\int_{0}^{l_{2}+l_{3}}xS\rho dx$$

$$T_{3}=\int_{0}^{l_{3}}xS\rho dx$$

where the $m_{o}^{\prime }$ is the integrity of end-effector and target object in normal gravity.

Therefore,

$$\tau_{1}^{\prime }=\tau_{2}^{\prime }\approx 0.8499\;N\cdot m=8.6667\;kg\cdot cm$$

$$\tau_{3}^{\prime }\approx 0.2490\;N\cdot m=2.5391\;kg\cdot cm$$

Based on the foregoing analysis, the motor selection requirements are summarized in the following table (Table 4).

Based on the above parameter requirements, multiple motors from different suppliers were compared, including specific models such as the ECX FLAT 22 S from Maxon, the DF20M012052-A from Nanotech, and the MS23HA0P4220-E from Moon’s Industries. Ultimately, the motor selected was the Pololu 1000:1 Micro Metal Gearmotor HP 6V with 12 CPR Encoder, Back Connector. This low-cost brushed DC motor from Pololu meets the dimensional requirements identified in the preceding analysis. It is equipped with a factory-matched 1000:1 reduction gearbox, capable of delivering the required torque output. In addition, it features a relative encoder, enabling the indirect determination of the motor’s angular position through time integration of the encoder signal.

### 2.5. Assembly of the Robotic Arm

**Figure 6:**
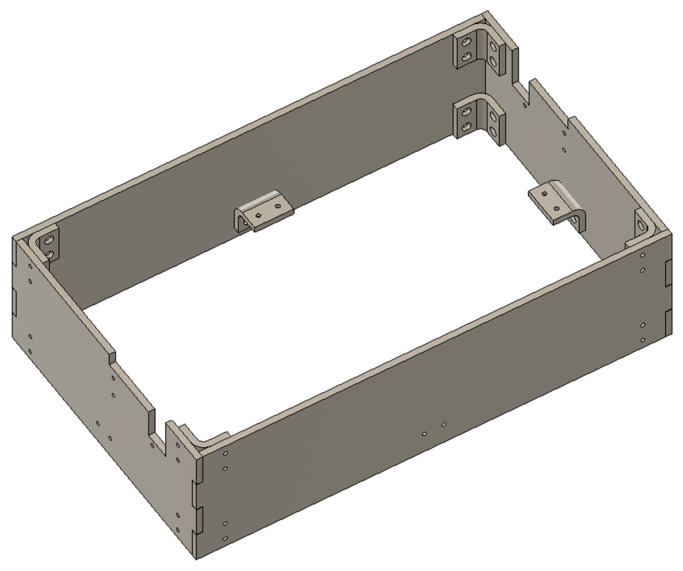
Hull assembly diagram.

To ensure the reliability of the panel connections, a custom connector was designed for joining laser-cut acrylic panels. This connector is fabricated using 3D printing and can be employed to join two acrylic panels or to attach an acrylic panel to a relatively flat surface of a 3D-printed component. The complete hull structure assembled with these connectors is shown in Figure 6.

**Figure 7:**
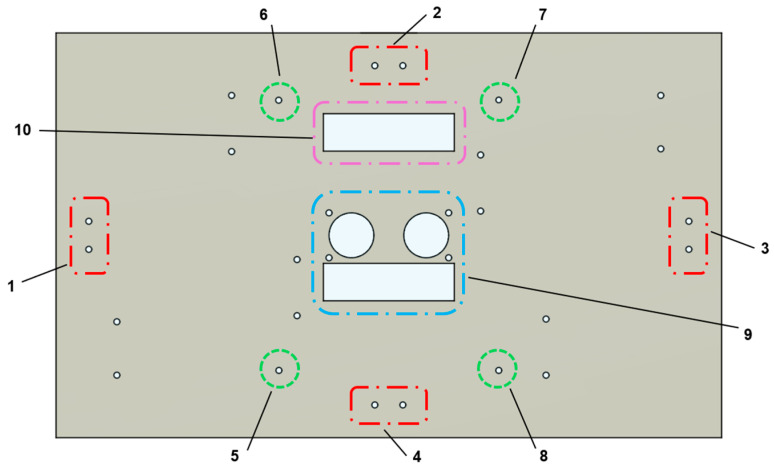
Hardware mounting panel hole layout diagram.

From the outset, the project was subject to a “compact design” requirement, stipulating that the entire robotic arm assembly must fit within a rectangular volume of 366 × 226.3 × 100 mm. Considering the functional requirements of the robotic arm and the dimensional constraints, this overall volume was divided into two compartments measuring 366 × 226.3 × 60 mm and 366 × 226.3 × 40 mm, respectively. The 60 mm-high compartment houses the robotic arm, while the 40 mm-high compartment accommodates the battery, circuit boards, cameras, and other hardware. These two compartments are separated by a mounting panel, on which all hardware components are installed. Following the same manufacturing approach as the hull, the mounting panel is fabricated from laser-cut acrylic and secured to the hull using the panel connectors described earlier. Mounting holes for the various hardware components are pre-designed into the panel. The layout of the panel and its mounting holes is shown in Figure 7.

**Figure 8:**
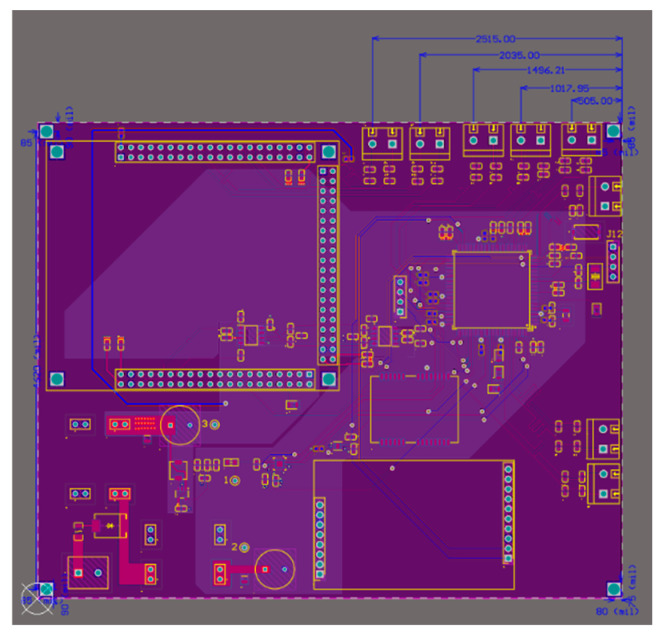
PCB designed by the hardware team.

Holes 1–4 correspond to the attachment points for the panel connectors, providing a secure interface between the panel and the hull through eight fastening points. Holes 5–8 are designated for mounting the PCB designed by the electronics hardware team (see Figure 8). Hole 9 is for mounting the binocular camera used for target recognition. For this purpose, the project employs the AN5642 camera module from Alinx as the stereo vision sensor. Hole 10 serves as a cable pass-through to the lower compartment.

**Figure 9:**
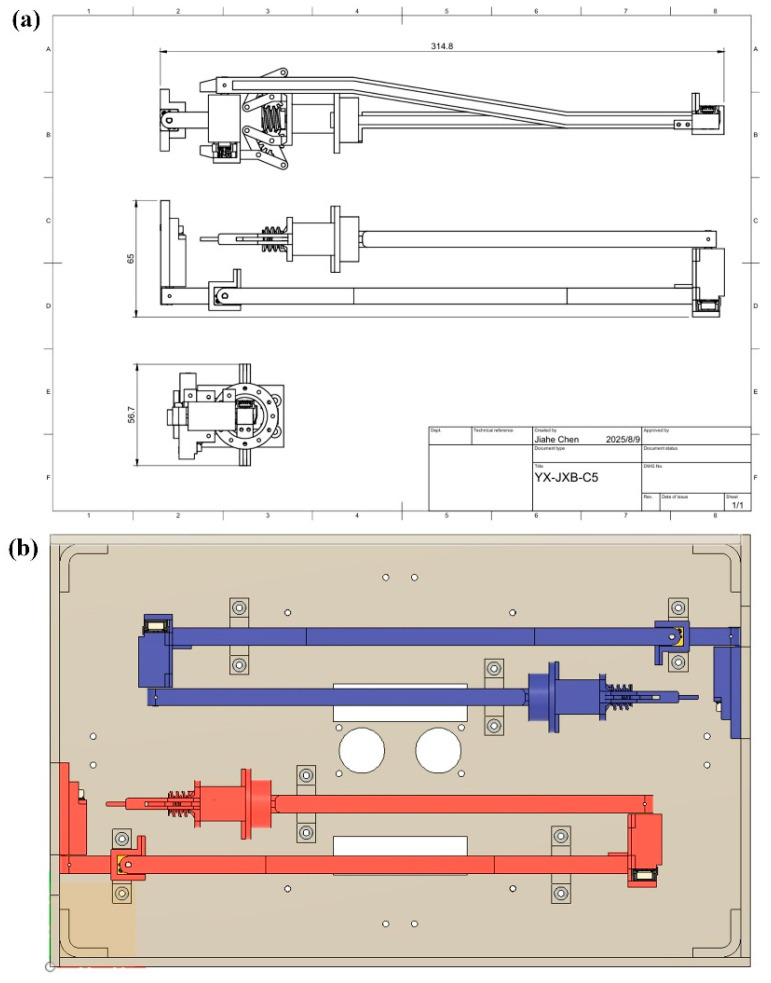
(**a**) Three-view drawings of the main structure of the robotic arm (with effector); (**b**) actual installation diagram (right).

After the design, manufacturing and selecting of each part, the overall assembling was conducted. Figure 9a illustrates the minimum installation dimensions for a single arm, measured at 314.8 × 65 × 56.7 mm, as indicated in the engineering drawing. The image in Figure 9b and Figure S1 shows the actual installation arrangement of both arms within the hull. In this configuration, the left and right arms are mounted facing each other, and their workspaces remain strictly separated when joint 2 is fixed. This ensures that no interference occurs during deployment or retraction of the arms. In the arrangement shown in the right image, the two arms are mounted in parallel, and the minimum installation volume does not exceed 314.8 × (65 × 2) × 56.7 mm, thereby satisfying the design constraint of 366 × 226.3 × 60 mm (Figure S2).

## 3. Verification of the 3-DOF Robotic Arm

Before manufacturing a robotic system, it is essential to verify its feasibility and performance through simulation. It is evident that most of the currently popular simulation tools are model-based. In practice, even a minor parameter modification requires the entire process of re-exporting the model, importing it into MATLAB (2024), verifying the imported parameters, and running the simulation. This workflow involves a considerable amount of repetitive work, which we want to avoid. Instead, the preference is to employ a “skeleton model” for limited functional verification of the robotic arm. Such a skeleton model would include only the essential parameters of the design, such as link lengths and joint orientations, along with the relevant mechanical properties of the materials composing the structure. In this work, we created a prototype simulation tool in Python (3.9) with the corresponding functionalities. All the code presented below is excerpted from the source code of the construction tool, which is available on GitHub Enterprise Server 3.17.7 [24].

### 3.1. Skeleton Model

In the simulation tool developed by the authors, the entire process follows a workflow of model configuration, analysis, control, and visualization. As illustrated in Algorithms 1 and 2, this depicts the construction process and trajectory planning of a robotic arm. In this case, the modeling of the arm does not require a complex and fully detailed three-dimensional model. Instead, it can be accomplished using only key parameters, analogous to a configuration file. The example pseudo code provided in this section generates the left arm of the dual-arm assembly designed in the present project. Existing tools, such as ROS MoveIt and PyBullet, typically require complete 3D model files. The skeleton model approach adopted in this work has advantages of simple and computationally efficient, which is suitable for high-frequency parameter optimization. This is the reason why this method was chosen for this work.
**Algorithm 1.** The construction process of a robotic arm.1:Create components objects (CO) using class ***Link***, ***RotationalJoint***, etc. These classes extend from *Part* and contains physical properties of each component such as density, length, direction, etc.2:Construct a robotic arm object (RAO) using components with ***.construct()***. This function will add CO to RAO’s ***parts*** property.3:Bind RAO to a controller object (CTRLO) such as ***SimulationController***. This kind of object will perform relative calculations and receive or input control signals into RAO.4:CTRLO executes. ***initialize()*** function to initialize bound RAO: set RAO to default states, retrieve ***torque_limit_dict***, etc.

**Algorithm 2.** Trajectory planning of a robotic arm.1:Input parameter to CTRLO: target pose matrix list ***L*** containing a series of target pose matrixes.2:Generate trajectory list using ***trajectory_generate_function*** with every pair of target pose matrixes in ***L***:3:
**Repeat**
4:    Obtain $P_{1}$, $P_{2}$ from ***L*** as start point and end point of a trajectory.5:    Execute ***check_reachable()*** to check if there is a solution in joint space from ***P1*** to ***P2***.6:    **If** reachable:7:        Obtain joint variables of each point ***control_variable_list*** in trajectory.8:        Calculate angular acceleration ω between each two control variables in ***control_variable_list***.9:        **If** exceed limitation $\omega_{limit}$:10:          Insert proper midway point to update ***control_variable_list***.11:Execute ***trajectory_input()*** with ***control_variable_list*** to generate and input control signal to bound RAO.

Based on the requirements of this project, three primary “model classes” were designed. The term “model class” refers to a set of classes used to store parameters of the skeletal model, *RoboticArm* is one such example. In addition, *Link* and *RotationalJoint*, which are utilized in constructing *RoboticArm*, are also considered “model classes.” All model classes inherit from a common base class named Assembly. On this foundation, *RoboticArm* implements an interface called *KinematicComputableAssembly*, which equips all *RoboticArm* instances with the capability for kinematic analysis. Similarly, all *RotationalJoint* classes implement the *Joint* interface, which provides each *RotationalJoint* instance with joint-related functionalities, such as adjusting the current joint input values of the assembly or retrieving the current joint outputs. Furthermore, the *Joint* interface and the *Link* class inherit from the common base class *Part*. Although the robotic arm designed in this project employs only rotational joints, the Joint interface has been designed with prismatic joints in mind. Consequently, if future applications require such joints, a *PrismaticJoint* class can be developed following the same design logic.

**Figure 10:**
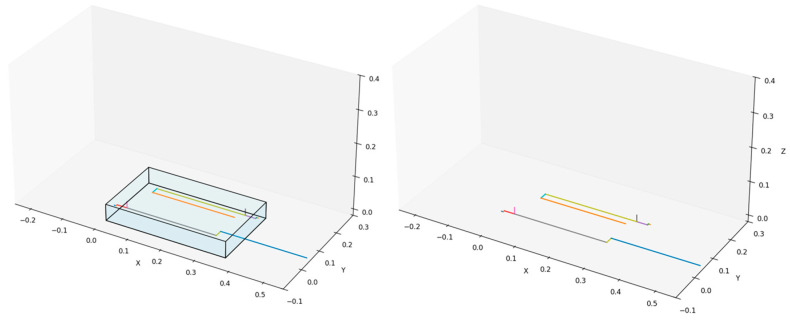
Rendered skeletal model of the robotic arm. The (**left panel**) illustrates the complete structure, including the storage space for the arm, while the (**right panel**) renders only the skeletal models of the left and right arms.

Adopting such a class hierarchy reduces coupling between component classes and enhances the maintainability of the project code. Furthermore, the *Assembly–Part* construction relationship clearly reflects the compositional structure of the assembly (Figure 10).

**Figure 11:**
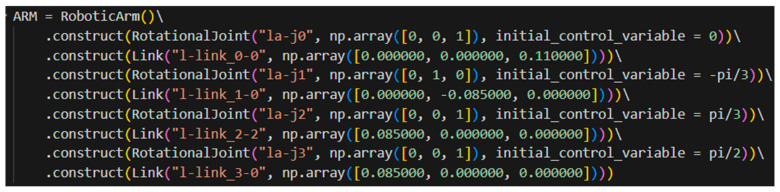
Construction of the skeletal model based on the Yahboom Dofbot.

As previously mentioned, the primary objective of the simulation tool developed in this study is to enable rapid iteration of the current design using skeletal models. This approach allows minor structural adjustments to be reflected by modifying several relevant numerical parameters in the component construction process, rather than repeating the full cycle of modeling–exporting–importing–adjusting. The following section presents a practical example to substantiate this feature of the simulation tool. The project team previously procured a Dofbot robotic arm from Yahboom as a verification platform for algorithm development [25]. The Yahboom Dofbot is a six-degree-of-freedom robotic arm, with two of its degrees of freedom dedicated to controlling the end-effector’s orientation. As illustrated in Figure 11, this code snippet demonstrates the construction of the skeletal model for the Yahboom Dofbot. It is evident that its configuration closely resembles the configuration code shown in Figure 10 for the custom-designed robotic arm in this project, with adjustments made only to the number of revolute joints, link lengths, link orientations, and joint axis directions.

**Figure 12:**
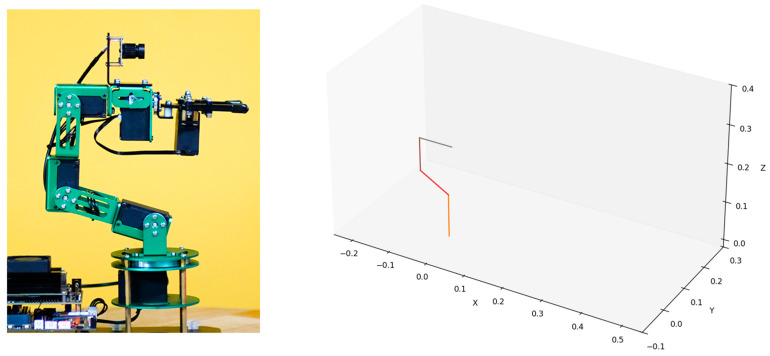
Physical view of the Yahboom Dofbot (**left**) and the skeletal model visualized in the simulation tool (**right**).

The left panel of Figure 12 presents the physical view of the Yahboom Dofbot, while the right panel shows the skeletal model of the robotic arm visualized in the same state as depicted in the left panel. All parameters of the above skeletal model are derived from the official 3D model files provided by Yahboom for the Dofbot.

It is evident that the skeletal model accurately reproduces the pose of the robotic arm in a given state and incorporates precise key parameters, such as joint inputs, orientations, and link lengths. Although the two robotic arms differ substantially in configuration design, the process of constructing their skeletal models is largely similar and equally efficient. At the same time, the skeletal model omits many unnecessary modeling details, enabling the designer to focus on the abstract structural design of the robotic arm itself. It should be noticed that the skeletal model merely defines a static abstract representation of a robotic arm configuration. If kinematic analysis of a given configuration or dynamic validation of joint outputs is required, a further automated analysis process must be performed.

### 3.2. Model Analysis

This section focuses on the mathematical analysis based on that skeletal model. Through mathematical analysis of the skeletal model, the simulation tool can automatically establish reference frames, generate the D-H table, and subsequently perform forward and inverse kinematic analysis.

The Denavit–Hartenberg (D-H) method is a systematic procedure for representing the kinematic structure of serial robotic manipulators. It provides a standardized convention for assigning coordinate frames to each link of a manipulator, thereby simplifying the derivation of the mathematical relationships between joint parameters and the spatial position and orientation of the end-effector. D-H analysis is essentially a process of reducing complexity to simplicity. For a complex robotic arm structure, it can produce a concise D-H table after analysis, upon which subsequent forward and inverse kinematic computations can be carried out efficiently. In the simulation tool developed in this study, however, a primary challenge lies in automating the D-H analysis and generating this table. As previously mentioned, the modeling process in this tool relies entirely on a set of key parameters specified when instantiating the assembly class. In other words, the problem transforms into determining how to construct the reference frame for the next revolute joint given the relative pose between two consecutive revolute joints, and then iteratively proceeding from the first joint to the last. This process, therefore, necessitates a degree of mathematical computation and derivation.

**Figure 13:**
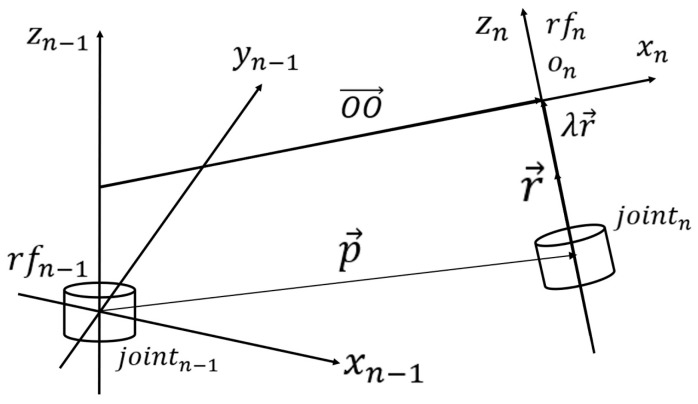
Schematic diagram of the abstract pose relationship between two adjacent revolute joints and their associated reference frames.

Figure 13 clearly illustrates the modeling of reference frames on two adjacent revolute joints in the general case. Since this represents a general situation, it cannot be assumed that the rotation axes are strictly parallel or perpendicular to each other. Under this condition, $x_{n-1}$, $y_{n-1}$, $z_{n-1}$ denote the reference frame attached to the preceding joint, while $x_{n}$, $y_{n}$, $z_{n}$ denote the reference frame attached to the adjacent subsequent revolute joint. These two reference frames are collectively referred to as ${rf}_{n-1}$ and ${rf}_{n}$. ${joint}_{n-1}$ and ${joint}_{n}$, respectively, represent the two adjacent joints themselves. $\vec{OO}$ is a vector perpendicular to the z-axes of both ${rf}_{n-1}$ and ${rf}_{n}$. $O_{n-1}$ and $O_{n}$ are the two intersection points of the $\vec{OO}$ vector with the z-axes of ${rf}_{n-1}$ and ${rf}_{n}$, respectively. It is evident that, according to the definition of the D-H method, the $\vec{OO}$ vector is in fact parallel to the x-axis of ${rf}_{n}$, and $O_{n}$ is actually the origin of ${rf}_{n}$. However, unlike the strict D-H convention, the origin of the reference frame on ${joint}_{n-1}$ is not located at the intersection of $z_{n-1}$ with the perpendicular from $z_{n-2}$ to $z_{n-1}$, but rather precisely at the physical position of ${joint}_{n-1}$ itself. This difference necessitates applying an offset along the z-axis in each step of the computation to eliminate the discrepancy. $\vec{p}$ is a vector pointing from the actual position of the preceding joint to the actual position of the subsequent joint. $\vec{r}$ is a unit vector originating from the subsequent joint and pointing in the direction of its rotation axis. λ is a scaling factor such that the end point of $\lambda \vec{r}$ coincides with $O_{n}$.

According to the definition of the skeletal model, for each segment in the computation, the known quantities include the vector $\vec{p}$ and the vector $\vec{r}$. These provide sufficient conditions to carry out the subsequent calculations. In the subsequent calculations, the definition of $a_{i}$, $\alpha_{i}$, $d_{i}$, $\theta_{i}$ remains unchanged.

Assume

$$\vec{p}=\left(x_{p},\;y_{p},z_{p}\right)$$

$$\vec{r}=\left(x_{r},\;y_{r},z_{r}\right)$$

We have

$$O_{n-1}=(0,\;0,\;d)=\vec{z}$$

According to the definition of the D–H method, d in this context corresponds to $d_{n}$, which is the displacement between the origins of two adjacent reference frames measured along the z-axis of the latter frame.

$$O_{n}=\vec{p}+\lambda \vec{r}=(x_{p}+\lambda x_{r},y_{p}+\lambda y_{r},z_{p}+\lambda z_{r})\;\vec{OO}=(x_{p}+\lambda x_{r},\;y_{p}+\lambda y_{r},\;{\;z}_{p}+\lambda z_{r}-d)\;∵\vec{OO}⊥\vec{r},\;\vec{OO}⊥\vec{z}\;∴\left\{\begin{array}{c} \vec{OO}\cdot \vec{r}=0\\ \vec{OO}\cdot \vec{z}=0 \end{array}\right.\;\Rightarrow \left\{\begin{array}{c} (x_{p}+\lambda x_{r})x_{r}+{(y}_{p}+\lambda y_{r})y_{r}+(z_{p}+\lambda z_{r}-d)z_{r}=0\\ (z_{p}+\lambda z_{r}-d)d=0 \end{array}\right.$$

Two cases arise here: the special case d=0, in which there is no offset between the two reference frames along the zzz-axis, and the general case with a nonzero offset. The analysis proceeds by considering these cases separately below.

case 1: d=0

$$\left\{\begin{array}{c} \lambda =-\frac{x_{p}x_{r}+y_{p}y_{r}+z_{p}z_{r}}{{x_{r}}^{2}+{y_{r}}^{2}+{z_{r}}^{2}}\\ d=0 \end{array}\right.$$

case 2 $\left(z_{p}+\lambda z_{r}-d\right)=0\;\Rightarrow d=z_{p}+\lambda z_{r}$

$$\left\{\begin{array}{c} \lambda =-\frac{x_{p}x_{r}+y_{p}y_{r}}{{x_{r}}^{2}+{y_{r}}^{2}}\\ d=z_{p}-\frac{x_{p}x_{r}+y_{p}y_{r}}{{x_{r}}^{2}+{y_{r}}^{2}}\cdot z_{r} \end{array}\right.\;∴a=\sqrt{{\left(x_{p}+\lambda x_{r}\right)}^{2}+{\left(y_{p}+\lambda y_{r}\right)}^{2}+{\left(z_{p}+\lambda z_{r}-d\right)}^{2}}\;\vec{x_{n-1}}=(1,\;0,\;0),\;\vec{z_{n-1}}=(0,\;0,\;1)\;\theta =∠(\vec{x_{n-1}},\vec{OO})\;\alpha =∠(\vec{z_{n-1}},\;\vec{OO})\;\vec{OO}=(x_{p}+\lambda x_{r},\;y_{p}+\lambda y_{r},\;{\;z}_{p}+\lambda z_{r}-d)$$

**Figure 14:**
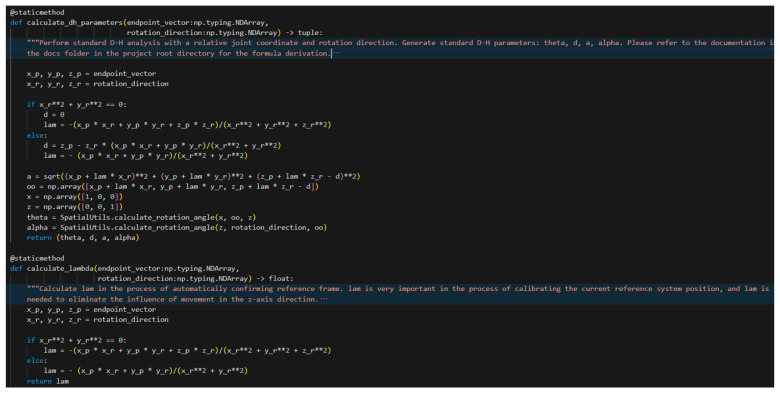
Implementation of the Automated D-H Analysis Process.

As noted earlier, from the construction of the skeletal model, the values of $\vec{p}$ and $\vec{r}$ can be determined. Consequently, substituting the values of $\vec{p}=\left(x_{p},\;y_{p},z_{p}\right)$ and $\vec{r}=\left(x_{r},\;y_{r},z_{r}\right)$ accordingly allows $a_{i}$, $\alpha_{i}$, $d_{i}$, $\theta_{i}$ to be computed. Additionally, in each segment of the computation, the origin of the reference frame on the preceding revolute joint exhibits a displacement along the z-axis, which must be eliminated during calculation. In this context, the offset is given by $\lambda \vec{r}$. The code implementation corresponding to the mathematical derivation presented above is shown in Figure 14.

Furthermore, the detailed derivation process of the D-H parameter table (Table 5) and the schematic diagram (Figure S3) of the manipulator with the D-H coordinate axes also have been described in the paper. This table presented here corresponds to the parameters of the left arm in its default configuration. The parameters of the right arm are the mirrored counterparts of those of the left one, with the remaining structure unchanged; therefore, they are omitted.

Following the mathematical derivations and code implementation presented above, the automated D-H analysis can be performed from the base to the final joint. Since the resulting D-H table depends on the current input of each joint, any change in the rotation angle of a joint motor will, in principle, cause a corresponding change in the D–H table. Consequently, the table is computed in real time and stored within the instance of *RoboticArm* class.

### 3.3. Pose Matrix Binding

In the previous section, an improved automated reference frame construction process based on the D-H method was introduced, with the final output of this process being the D-H table. In this section, the discussion will continue with the use of the D-H table obtained in the previous section to derive and store the overall state of the robotic arm. In this work, the analysis performed by the developed simulation tool focuses on the so-called “skeletal model.” Consequently, in the subsequent analysis process, attention is directed toward the orientation of each component (e.g., the rotation direction of a revolute joint) and the positions of its start and end points (e.g., for a link). As a result, the overall state of the robotic arm can instead be stored in terms of these key parameters. Since the complete D-H parameter table can be obtained through the mathematical computations described earlier, it becomes possible to determine the pose matrix of each reference frame in the world coordinate system using forward kinematic analysis. It is important to note that, in the earlier construction of the “skeletal model,” all pose parameters were defined relative to the preceding component. Consequently, it follows that, once the pose matrix of the preceding component is known, the pose matrix of the subsequent component can be correspondingly derived.

**Figure 15:**
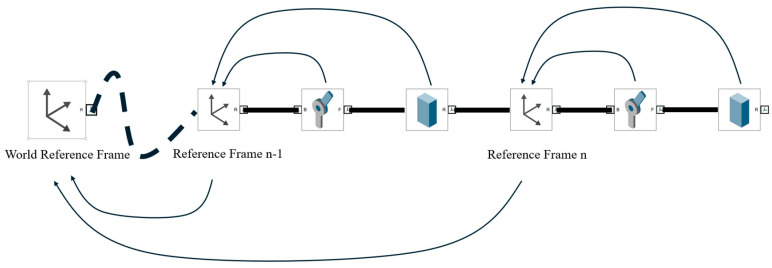
Schematic of the pose-matrix derivation process for the individual components of the robotic arm.

As shown in Figure 15, unlike the conventional D-H method, although the origin of each reference frame is located on the corresponding joint’s axis of rotation, an instance of *RoboticArm* not only records the position of the reference frame origin in the world coordinate system, but also stores the offset of each joint relative to the z-axis of its own reference frame. This offset, as derived in the earlier mathematical analysis, corresponds to $\lambda \vec{r}$. In addition, this schematic illustrates a segment with a joint–link–joint–link structure, in which each joint is associated with a corresponding reference frame. Starting from the world coordinate system and using the D-H table generated through the automated process, the pose matrices of reference frame ${reference\;frame}_{n-1}$ and ${reference\;frame}_{n}$ in the world coordinate system can be obtained. Furthermore, based on the earlier mathematical derivations, the pose matrix of each joint relative to its associated reference frame can be recovered. On this basis, the transformation matrices from each component of the instance of *RoboticArm* to the preceding joint can be conveniently obtained from the component parameters. For example, for a Link component, the pose matrices of both its start and end points (relative to the reference frame on the preceding joint) can be determined. Through this series of transformations, the pose matrix of any point on the robotic arm at any moment, relative to the world coordinate system, can be obtained by composing two transformations: from the base point to the reference frame of the corresponding joint, and from that reference frame to the target point.

**Figure 16:**
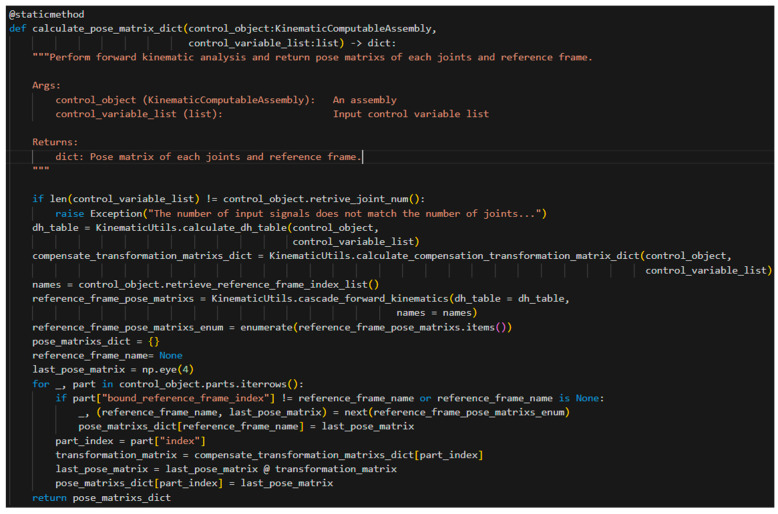
Deriving the pose matrices of the start and end points of each component from D-H table.

Figure 16 presents the code implementation of the process described above. Each time a joint motor is actuated, such a computation is performed to update the pose information of the robotic arm stored within the instance of *RoboticArm*.

### 3.4. Workspace Analysis

**Figure 17:**
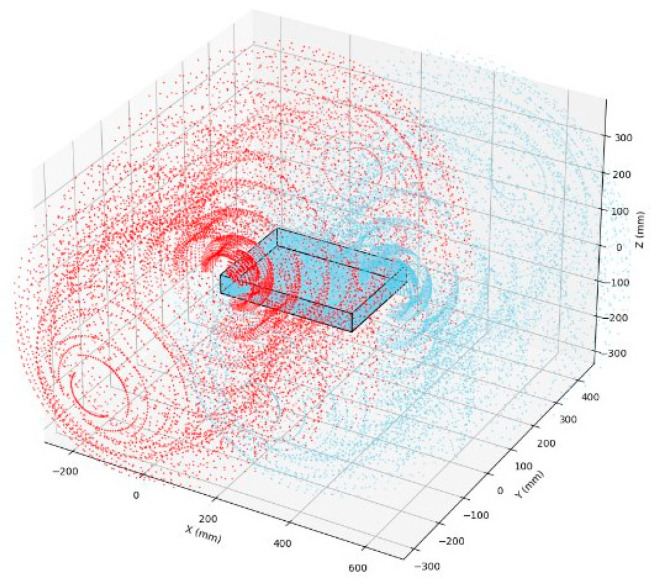
Point cloud of theoretically reachable positions within the workspace.

**Figure 18:**
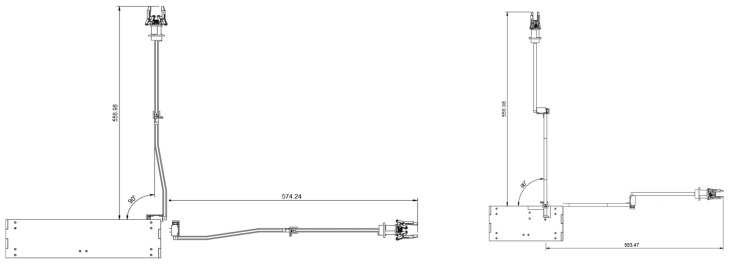
Boundary position of the right manipulator in the front view and right view.

In the workspace analysis of the robotic arms, a point cloud approach was adopted as the analytical tool. Since each robotic arm possesses three degrees of freedom, the joint space of each actuated revolute joint was sampled at intervals of 5°, resulting in the workspace point cloud illustrated in Figure 17. In this figure, the red points represent the reachable positions of the left arm’s end-effector, while the blue points indicate those of the right arm’s end-effector. It can be observed that both the left and right arms exhibit a relatively large theoretical workspace, which aligns well with the operational objective of grasping target objects. Figure 17 presents the maximum reachable positions of the arms in the actual case, as viewed from the front and right. However, it should be noted from Figure 18 that a dead zone exists in the vicinity of the hull structure, implying that the arms cannot reach positions that are too close to the base. Targets must be located outside this dead zone in order to be grasped by the arms, a factor that should be taken into careful consideration in subsequent algorithm design.

Overall, excluding the region along the negative z-axis in Figure 17 that is unreachable due to obstruction by the hull structure, the workspace of a single arm exhibits an approximately hemispherical shape, which is consistent with the initial design. Further computation of the workspace will be carried out by the algorithm team and will not be elaborated upon in this paper.

### 3.5. Interference Analysis Between Arms

This section is limited to determining whether interference occurs between the left and right arms during deployment, and whether the arm interferences either itself or the hull during this single deployment motion. It should first be noted that, since the design of the end-effector for this project was provided by another team, the finalized end-effector design is directly adopted here without elaborating on its development process.

Based on the minimum installation volume discussed, it is evident that, in the current design, the mounting points of the left and right arms exceed the width of the minimum installation volume. Therefore, by simple geometric reasoning, interference between the left and right arms during deployment can be readily excluded. In addition, the design drawings reveal two boundary conditions where interference is most likely to occur: first, during the deployment of Link 3, the tip of the end-effector may come into contact with the motor housing of Joint 1; second, when the output of Joint 1 approaches 90°, the fixed structure of Joint 2 may potentially interfere with the hull.

**Figure 19:**
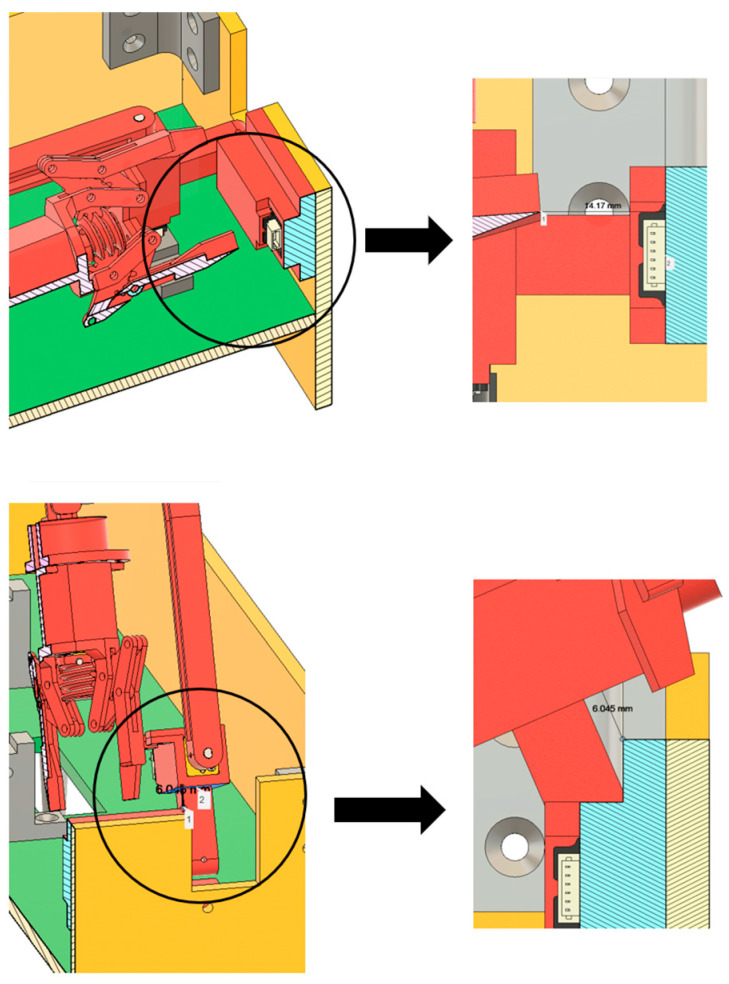
Cross-sectional analysis of the arm’s deployment process under boundary conditions.

Related cross-sectional analysis of the arm’s deployment process, as is shown in Figure 19, and extensive geometric computation further infer that, under boundary conditions mentioned earlier, the end-effector does not interfere with the arm’s own structure nor hull framework, with enough clearance margin still maintained. This outcome demonstrates the correctness of the relevant parameter calculations carried out during the design process.

## 4. Conclusions

The design and validation of this compact 3-DOF dual-arm robotic system successfully address the core objectives of modularity, cost-effectiveness, and compactness. This article highlights three core innovative points: adopting orthogonal joint configuration and modular structure to achieve compact folding size and hemispherical workspace; by mixing manufacturing technology, the system cost is controlled; a Python-based skeleton modeling tool that supports automated D-H parameter generation and rapid design iteration is developed.

In this work, the final assembly achieves a stowed configuration within the target dimensions of 366 × 226.3 × 100 mm, fulfilling the stringent size constraints while maintaining functional versatility. The orthogonal joint configuration and reduced motor count (three per arm) balance workspace requirements with budgetary limits (£2000), leveraging hybrid manufacturing (laser-cut acrylic hull and 3D-printed ASA-CF arm components) to optimize cost and performance. Kinematic validation via the custom “skeleton model” simulation tool confirmed the design’s feasibility. The point cloud analysis revealed a hemispherical workspace for each arm, suitable for grasping and transferring 50 mm targets, though a dead zone near the hull necessitates algorithmic compensation. Interference analysis verified collision-free deployment under boundary conditions, while finite-element analysis identified tolerable deflection (5–20 mm) under load (Figure S3), mitigated by stereo vision calibration. Future work should address fabrication tolerances in 3D-printed parts and explore alternative link geometries to reduce deflection. The modular design framework, however, provides a scalable foundation for applications ranging from satellite servicing to terrestrial robotics, emphasizing adaptability to diverse environments. By integrating lightweight materials, simplified kinematics, and rapid prototyping, this project advances the development of accessible, space-efficient robotic systems for non-industrial settings. For example, a low-cost and simple 3-DOF arm robotic system is suitable for students to create visual grasping systems and achieve rapid development in non-ROS environments through Python programming.

## Figures and Tables

**Table 1:** Comparison of common manufacturing methods.

Manufacturing Method	Typical Precision	Material Compatibility	Relative Cost	Advantages	Limitations
CNC Machining	±0.02–0.05 mm	Metals, plastics, composites	High	High precision, excellent surface finish, strong mechanical properties	High cost, long lead time, less flexible for rapid design changes
Laser Cutting	±0.1–0.2 mm	Acrylic, plywood, thin metals, composites	Low–Medium	Fast, cost-effective for flat sheet cutting, high repeatability	Limited to 2D geometries, thickness constraints
FDM 3D Printing	±0.2–0.5 mm	PLA, ABS, ASA, PETG, composites (e.g., carbon-fiber-filled)	Low	Complex geometries, no tooling required, rapid prototyping possible	Lower precision, anisotropic mechanical properties, post-processing often needed

**Table 2:** Comparison of common 3D printing materials.

Material	Typical Extrusion Temp.	Key Advantages	Limitations
PLA	~180–230 °C	Easy to print, biodegradable	Low heat resistance, brittle
ABS	~210–250 °C	Durable, impact-resistant	High warping, fumes, moderate UV resistance
ASA	~240–260 °C	UV/weather resistant, high impact & heat resistance	Requires high temp enclosure, emits styrene fumes
PETG	~220–235 °C	Flexible, durable, easier than ABS	Lower stiffness, moderate heat resistance

**Table 3:** Technical data sheet of ASA-CF material.

Property	Test Standard	Data (XY)	Data (Z)
Young’s Modulus	ISO 527, GB/T 1040	4200 ± 270 MPa	2290 ± 260 MPa
Tensile Strength	ISO 527, GB/T 1040	34 ± 3 MPa	30 ± 4 MPa
Elongation at Break	ISO 527, GB/T 1040	9.6 ± 1.4%	4.4 ± 0.8%
Flexural Modulus	ISO 178, GB/T 9341	3740 ± 130 MPa	1350 ± 120 MPa
Flexural Strength	ISO 178, GB/T 9341	72 ± 5 MPa	33 ± 3 MPa
Impact Strength	ISO 179, GB/T 1043	14.0 ± 2.2 kJ/m^2^	9.4 ± 0.6 kJ/m^2^
Notched Impact Strength	ISO 179, GB/T 1043	6.2 ± 1.4 kJ/m^2^	N/A

**Table 4:** Motor selection requirements table.

Torque Output		Price Limitation (£)		Frame Diameter (mm)	Shaft Cross-Section Shape
N·m	kg·cm				
0.8499	8.6667	<100	20–50		D-shape

**Table 5:** The detailed derivation process of the D-H parameter table.

	D-H Parameters Table			Unit: Meter
	θ	d	a	α
Joint 1	0	0	0	0
Joint 2	0	0	0.0051	$-\frac{\;\pi }{2}$
Joint 3	0	0.0053	0.3089	$\frac{\;\pi }{2}$
Joint 4	0	0	0.2699	$-\frac{\;\pi }{2}$

## Data Availability

The original contributions presented in this study are included in the article/Supplementary Material. Further inquiries can be directed to the corresponding authors.

## Supplementary Materials

The following supporting information can be downloaded at https://www.mdpi.com/article/10.3390/s25237356/s1, Figure S1. Rendered diagram of the assembly of robotic arm and hull. Figure S2. Three-view drawing of the assembly. Figure S3. The schematic diagram of the manipulator with the D-H coordinate axes. Figure S4. Simple displacement analysis of Link 2 under target load.
