# Supplementary material for: A Mechanical Structure Design and Simulation-Based Validation of a Novel Compact and Low-Cost 3-DOF Robotic Arm
Source: Sensors (Basel). 2025 Dec 3;25(23):7356. doi: 10.3390/s25237356 (PMC12694365; doi:10.3390/s25237356)
Supplement: Supplementary file 1 [file sensors-25-07356-s001.zip › sensors-3956010-supplementary.pdf]

A Mechanical Structure Design and Simulation-Based Validation of a Novel Compact and Low-Cost 3-DOF Robotic Arm

Jiahe Chen [2], Bojun Jiang [3], Shu Zhu [1,2,3,*] and Jun Wang [1,2,*]

[1] Wenzhou Key Laboratory of Al Agents for Agriculture, Wenzhou 325006, China
[2] Wenzhou Vocational College of Science and Technology, Wenzhou 325006, China; fut.streich@outlook.com
[3] Jiangsu Key Laboratory of 3D Printing Equipment and Manufacturing, School of Electrical and Automation Engineering, Nanjing Normal University, Nanjing 210023, China; 13013912211@163.com
* Correspondence: shu.zhu@nnu.edu.cn (S.Z.); jonwang_paper@163.com (J.W.)

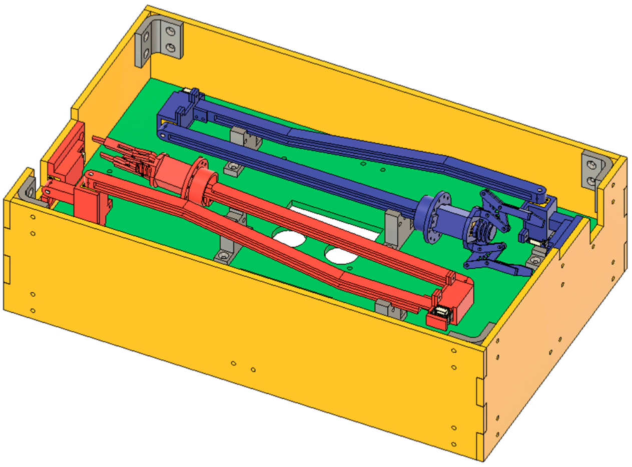

Fig.S1. Rendered diagram of the assembly of robotic arm and hull.

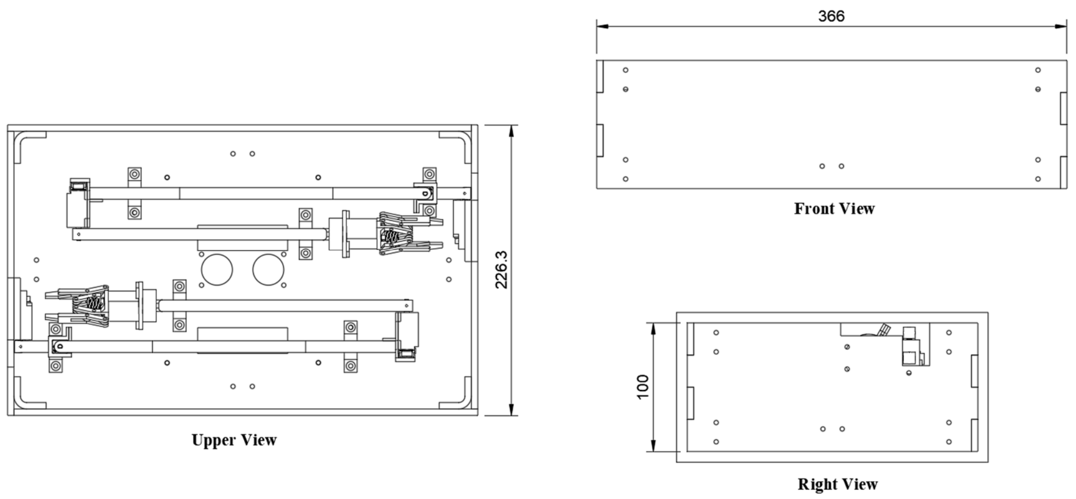

Fig.S2. Three-view drawing of the assembly.

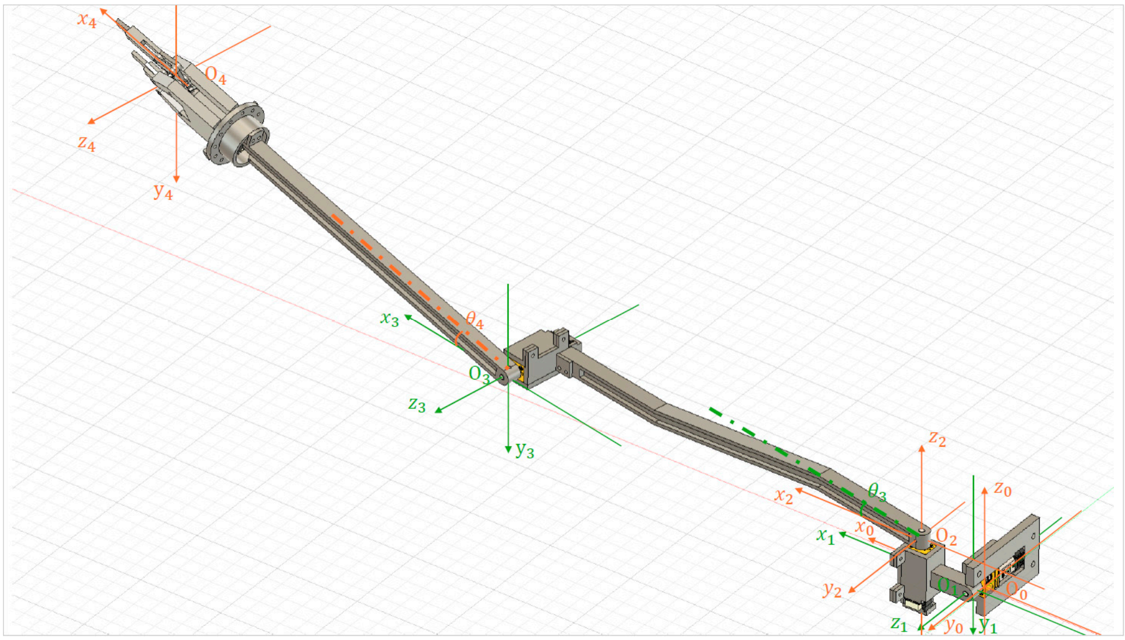

Fig.S3. The schematic diagram of the manipulator with the D-H coordinate axes.

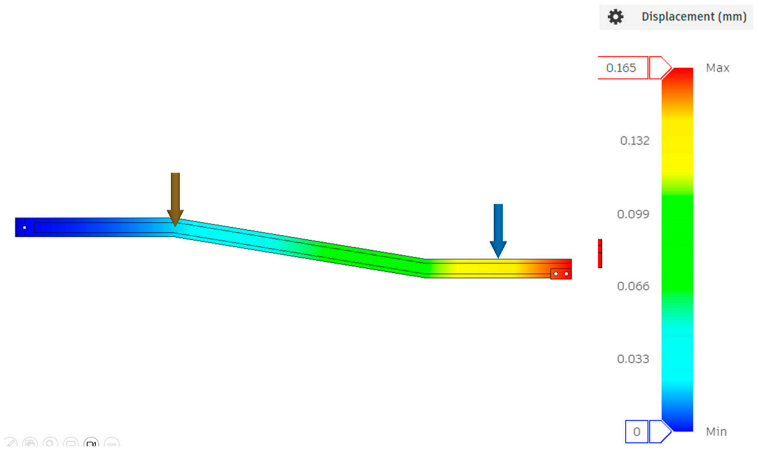

Fig.S4. Simple displacement analysis of Link 2 under target load. The hardware design and verification aspects of this project have largely achieved the predetermined objectives, though certain details require further investigation in the future. For example, in the finite element analysis of a single arm, a relatively large deflection was observed when simulating the application of the target mass at the arm's end. Although the magnitude of this deflection remains within an acceptable range, it can still introduce errors in end-effector position calculation. As shown in Fig. S4, although the end displacement under the target load is almost negligible, the fully extended configuration of the arm amplifies this error. At present, the error consistently remains within the range of 5–20 mm.
